# Major QTLs for Trunk Height and Correlated Agronomic Traits Provide Insights into Multiple Trait Integration in Oil Palm Breeding

**DOI:** 10.3390/genes11070826

**Published:** 2020-07-21

**Authors:** Chee-Keng Teh, Ai-Ling Ong, Sean Mayes, Festo Massawe, David Ross Appleton

**Affiliations:** 1 Biotechnology & Breeding Department, Sime Darby Plantation R&D Centre, Serdang 43400, Selangor State, Malaysia; ong.ailing.sdtc@simedarbyplantation.com (A.-L.O.); david.ross.appleton@simedarbyplantation.com (D.R.A.); 2 School of Biosciences, University of Nottingham Malaysia, Semenyih 43500, Selangor State, Malaysia; festo.massawe@nottingham.edu.my; 3 School of Biosciences, University of Nottingham, Sutton Bonington Campus, Leicestershire LE12 5RD, UK; Sean.Mayes@nottingham.ac.uk

**Keywords:** compact palms, marker-assisted selection, *Elaeis guineensis*, linkage maps, dwarfism

## Abstract

Superior oil yield is always the top priority of the oil palm industry. Short trunk height (THT) and compactness traits have become increasingly important to improve harvesting efficiency since the industry started to suffer yield losses due to labor shortages. Breeding populations with low THT and short frond length (FL) are actually available, such as *Dumpy* AVROS *pisifera* (DAV) and *Gunung Melayu dura* (GM). However, multiple trait stacking still remains a challenge for oil palm breeding, which usually requires 12–20 years to complete a breeding cycle. In this study, yield and height increment in the GM × GM (GM-3341) and the GM × DAV (GM-DAV-3461) crossing programs were evaluated and palms with good yield and smaller height increment were identified. In the GM-3341 family, non-linear THT growth between THT_2008 (seven years old) and THT_2014 (13 years old) was revealed by a moderate correlation, suggesting that inter-palm competition becomes increasingly important. In total, 19 quantitative trait loci (QTLs) for THT_2008 (8), oil per palm (O/P) (7) and FL (4) were localized on the GM-3341 linkage map, with an average mapping interval of 2.01 cM. Three major QTLs for THT_2008, O/P and FL are co-located on chromosome 11 and reflect the correlation of THT_2008 with O/P and FL. Multiple trait selection for high O/P and low THT (based on the cumulative effects of positive alleles per trait) identified one palm from 100 palms, but with a large starting population of 1000–1500 seedling per cross, this low frequency could be easily compensated for during breeding selection.

## 1. Introduction

Oil palm (*Elaeis guineensis* Jacq.) is a major source of vegetable oils and fats, contributing 35% of global vegetable oil production [1]. Per hectare of cropland, oil palm is able to yield 10 times more oil per year than any other temperate oil crop, which leads to oil palm cultivation being one of the most efficient agricultural land uses in humid tropical environments [2]. This also explains why only 7% of total global oilseed-harvested land is occupied by oil palms [3] yet ~35% of the global trade is in palm oil. To sustain long-term profit, enormous efforts have been put into improving oil yield per unit area, which involves two main components, i.e., fresh fruit bunch (FFB) production and oil-to-bunch ratio (O/B).

In recent years, an acute labor shortage in Malaysia has become a major problem for collecting FFB and loose fruit in the field. Foreign labor represents over 70% of the total 491,339 workforce of oil palm plantations in Malaysia, which reflects their high dependency on migrants who mainly come from Indonesia, Bangladesh, the Philippines, Thailand and Myanmar [4]. In addition, foreign workers are increasingly choosing to work in oil palm plantations in their home countries for similar or higher wages. This has contributed to stagnant oil yield in Malaysia, which leads to suboptimal estate management, even though high-yielding commercial seeds are available in the country. For example, Sarawak alone incurred losses of up to US$ 240 million per year in uncollected FFB because of labor shortages ranging from 15% to 20% across plantations [5]. Several mechanization approaches to harvest FFB and to collect loose fruit have been tested, but their commercial applicability is still restricted mainly due to the non-uniform palm architecture and topography of estates. Hence, the oil palm industry is keen to integrate oil yield traits and secondary vegetative traits, such as reduced trunk height and increased bunch stalk length [6] through selective breeding for better harvesting efficiency. Compact palms are a typical example. By retaining equivalent levels of inter-palm competition, the short rachis trait allows up to 180 palms per hectare, which is higher than the standard densities (138–143 palms per hectare) [7]. Improvements in yield per area, however, can only be fully realized with an increase in labor supply and/or increased levels of mechanization. Thus, the integration of slow height increment into compact palms is crucial to reduce the additional demand for labor and to facilitate mechanization.

One of the important genetic resources for reduced height increment is a self-pollinated family, Deli *dumpy* E206, which has been found to be uniformly short, indicating that the trait is highly heritable [8]. The family was crossed into many breeding programs, especially in Southeast Asia. In oil palm, the mesocarp oil content is partly determined by the presence/thickness of non-productive kernel shell in the fruit. To achieve 30% more oil, the commercial thin-shelled *tenera* are produced from crosses between thick-shelled *dura* and shell-less *pisifera* (usually female sterile). Hence, Deli *dura* with 75% *dumpy* ancestry × commercial AVROS *pisifera* produced a comparable oil yield to the *dura* × *pisifera* (DxP) control, while retaining lower trunk height growth due to the dumpy character [9]. Breeders from the Golden Hope and Highlands Research Unit (now under the Sime Darby Plantation) began exploiting this material differently. They introgressed the current pollen source, which is derived from AVROS *pisifera* and used very widely in Malaysia, into a new origin including 25% *dumpy* ancestry, termed *dumpy* AVROS, and hybridized these with normal Deli *dura* to generate commercial *tenera* palms with shorter trunk height increment [10]. Also, the company adopted the *Gunung Melayu dura* (GM), which originated from the Deli and was selected for its low height increment and high mesocarp oil content. No significant difference in trunk height between *GM* × AVROS *pisifera* and other Deli *dura* × AVROS *pisifera* was observed [11]. However, to date, evaluation of the hybridization between GM and *dumpy* AVROS *pisifera* (DAV) to reduce trunk height has yet to be reported.

The breeding fitness of a cultivar may drop when introgression from non-elite sources of germplasm occurs. The phenomenon is mostly due to linkage drag of the undesirable effects (normally from the wild donor) of non-targeted genes or quantitative trait loci (QTL) linked to the targeted genes or inherited from other chromosomes during crossing. Multiple selection and backcrossing cycles with sufficient progeny are crucial to identify the individuals that break the linkage drag by recombination between the QTL and undesirable genes to recover an elite background. Breaking of unwanted linkages in perennial crops, such as oil palm, based on phenotypic selection is costly, time consuming and inaccurate for all but the simplest phenotypes. Many fruit agronomic traits of oil palm can only be measured after three years of field planting, followed by another four years of recording to complete a selection cycle. In addition, the environmental effects on polygenic/complex traits, including oil yield and trunk height (THT) are strong, which presents a great challenge to capturing the genetic effects in improved breeding materials. To address this, locating the QTL underlying these traits has become increasingly important. The objectives of this study include: (1) to evaluate the trait variation in reduced trunk height increment between a short-trunk maternal GM × GM population and its *tenera* progeny (GM × DAV), (2) QTL mapping for THT and THT-correlated agronomic traits in a GM × GM population using a high-density OP200K genotyping array [12] and simple sequence repeats (SSRs), and (3) consolidating the QTL for selected traits and marker-assisted selection (MAS) to accelerate reduced height breeding programs with better trait predictability.

## 2. Materials and Methods

### 2.1. Plant Materials and DNA Preparation

A full-sib *dura* family, namely, GM-3341 (*n* = 113 palms) was obtained from a cross between GM-Parent1 and GM-Parent2. To determine variation in the THT trait, a progeny test family, i.e., GM-DAV-3461 *tenera* (*n* = 70 palms) derived from the common GM-Parent2 *dura* and DAV-Parent1 *pisifera* was selected and compared. Both GM-3341 and GM-DAV-3461 families were planted in 2001 in a randomized complete block design (RCBD) at a density of 148 palms per hectare in the East Estate (PT132 trial, 10 replicates per family) and the North Estate (PT138 trial, 5 replicates per family) of the Sime Darby Plantation on Carey Island, Malaysia. A total of 76 commercial DxP palms derived from an admixed parentage of Deli *dura* × AVROS *pisifera* were planted as a control in each trial. The control planted in the PT132 trial and PT138 trial were named DxP control (GM) and DxP control (GM-DAV), respectively. The estates generally share similar annual temperatures and rainfall. For linkage analyses, a total of 113 palms of the GM-3341 family were sampled at unopen frond 0 for DNA extraction. Total genomic DNA was isolated from 0.1 g of the leaf tissue using the DNAeasy Plant Mini Kit (Qiagen, Hilden, Germany). The DNA quality was visualized on a 0.8% agarose gel and quantified using the Nanodrop 2000 (NanoDrop Technologies, Wilmington, DE, USA). The DNA samples were stored at −20 °C in a freezer for subsequent genotyping.

### 2.2. Trait Recording and Statistical Analyses

In oil palm breeding (and research) programs, the industry standard is followed, which usually involve the planting of controlled crosses in RCBD in two locations. Crosses are extensively recorded for certain traits. For example, bunch yield for every bunch (usually one bunch per month) for several years, while others are more time intensive (such as bunch analysis, where a minimum of 3 per palm is standard) or have been deemed of less importance (height increment is one of these). Overall, this tends to reflect the ease of data collection as opposed to the importance of the trait for the breeding program [13]. In this study, yield recording was started when both trials reached three years old. Both the number of bunches (BNO) and average bunch weight (BWT) were recorded to generate 4-year averages for FFB (from 2004 to 2008). At least three bunches per palm were analyzed for O/B and other bunch components according to the industry standard [14] with modifications [15]. The total oil per palm (O/P) was then calculated from the multiplication of the 4-year average FFB and average O/B. For the THT trait, the distance from the ground to the 41st frond was measured in 2008 (as THT_2008) and 2014 (as THT_2014) using an extendable ruler. At the same time, other vegetative traits were also measured based on industry standard methods [16]. A total of 16 traits (Table 1) were compiled for standard descriptive statistics; trait values between 1.5 and 3 times the interquartile range were identified as outliers and removed. Presence of low THT and short frond length (FL) were determined separately in the GM-3341 family at the PT132 trial and the GM-DAV-3461 family at the PT138 trial by comparing them to their respective DxP controls using a two-sample *T* test at *p* = 0.05 threshold. A Pearson correlation analysis of the 16 traits in the GM-3341 family was also performed at the *p* = 0.05 threshold. All statistical tests were done in the Minitab^®^ 17 program [17] and box plots were generated in R.

### 2.3. DNA Marker Genotyping

A *tenera* palm, namely, Progeny palm 5002 derived from D4-93/76 (Deli *dura*) × 2/2311 (EKONA *pisifera*) was whole-genome sequenced with 30X coverage using the Roche 454 Titanium FLX platform. From the reference genome, a total of 11,653 SSRs were discovered and primers designed. The SSR amplification conditions with fluorescent-labeled M13-tailed forward primers [18] were optimized for each of the primer pairs and followed by genotype scoring using an Applied Biosystems 3730xl DNA Analyser (Applied Biosystems, Carlsbad, CA, USA). The SSR primers formed a multiallelic marker framework. Furthermore, the OP200K Infinium array (Illumina, San Diego, CA, USA) based on 59 breeding origins of oil palms [12] was applied to the population. Only the markers with heterozygous genotypes in at least one of the parents of the GM-3341 family were defined as informative and were used to genotype the mapping population.

### 2.4. Construction of Linkage Maps

The GM-3341 family, which is defined as a cross-pollinator (CP) population, was deployed as a mapping population. A full-density GM × GM linkage map was constructed using Lep-MAP3 [19,20] for large number of SNPs, which is applicable to outbred families, such as oil palm, based on the maximum likelihood method. Marker quality was checked under the *Filtering* module. Informative SNPs with >5% of missing data and/or identical genotype segregation were removed. Subsequently, the *Separate Chromosomes* module was used for binning the assayed SNPs in each parent based on the optimized logarithm of odds, to the base 10 (LOD) thresholds ranging from 4 to 30. To order the binned SNPs, the Kosambi mapping function was adopted for the conversion of recombination frequencies into map distances (in unit cM). The maps of both parents were joined as one using the option “sexAveraged = 1”. A subset of SNPs was selected from every 5 cM of the full-density linkage map and loaded into the Joinmap^®^ 5 program [21] for linkage mapping to enable QTL mapping analysis. In addition, a set of 67 informative SSRs were also included. The markers with segregation distortion at chi-square *p* < 0.001 (***) were removed. The selected markers were ordered based on optimized LOD thresholds (6.0–9.0) with the Kosambi map function and regression model, and the linkage map was visualized using the Mapchart 2.32 program [22]. Each LG number was identified with a chromosome of the published physical map [23]. In addition, the linked SNPs were also mapped onto the same physical map to estimate correlation between linkage (in cM) and physical (in Mb) positions in the genome (Tables S1 and S2), which allowed recombination estimates (in cM/Mb) across the pseudo-chromosomes. Finally, the average mapping interval was calculated at LG and genome-wide levels.

### 2.5. Quantitative Trait Locus Mapping Analyses

All QTL mapping analyses for THT_2008, THT_2014, O/P and FL traits were carried out using the MapQTL^®^ 6 program [24]. Initially, single marker tests using the rank sum test of Kruskal-Wallis (KW) were done at the *p* = 0.005 threshold, followed by interval mapping (IM) to identify potential QTL peaks. In order to determine the genome-wide LOD threshold, permutation tests were performed with 1000 iterations at α = 0.05, but ‘putative’ QTLs at 1.5 below the genome-wide LOD = 4.0 threshold were still reported. The significant QTLs detected in IM were fixed as cofactors for multiple QTL mapping (MQM) mapping. Any new QTLs detected were considered as cofactors for the next round of MQM mapping and the process were iterated until no change occurred. Then, trait variation explained by the identified QTL was determined based on MQM mapping analysis.

### 2.6. Cumulative Effects of Quantitative Trait Allele Analysis

The putative and genome-wide significant QTLs were selected to measure the marker effects for THT_2008, FL and O/P traits based on median comparison among alleles in Minitab^®^ 17 program [17]. In each QTL, the positive alleles in each trait that benefit the breeding program were defined in the GM-3341 family (107 palms after outlier removal). The alleles that decreased FL and THT_2008 values were known as positive, whereas the positive alleles for O/P increased the trait value. Next, the total number of positive alleles across the QTLs in individual palms were plotted against each trait to determine the cumulative effects of quantitative trait alleles. The threshold number of positive alleles for each trait selection was defined based on the intersection between the mean line of a trait and the fitted line on a scatter plot. The threshold numbers of positive alleles and mean trait values were then used to perform palm selection for multiple traits on a scatter plot between traits.

## 3. Results

### 3.1. Descriptive Statistics and Comparison of Traits between Families

**Figure 1:**
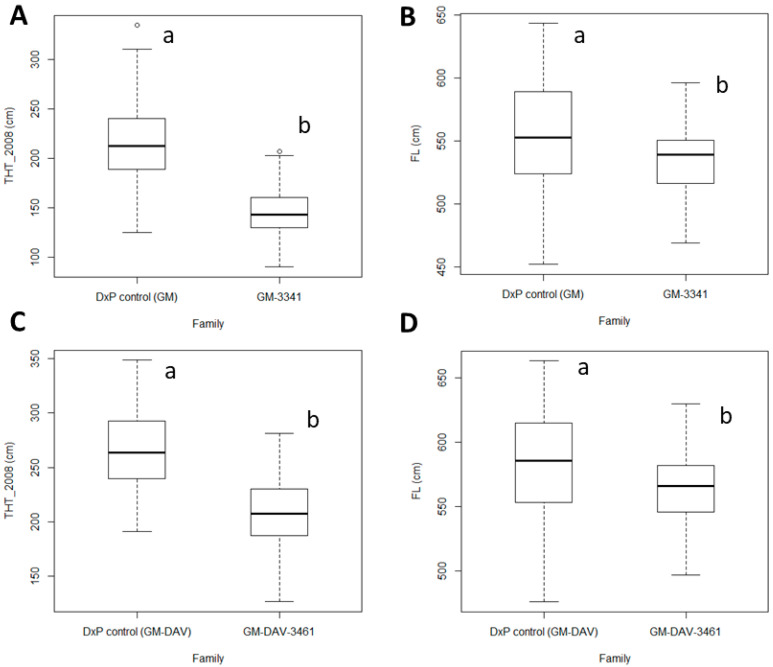
The boxplots represent median values, percentile 25–75 for trunk height measured in 2008 (THT_2008) and frond length (FL) between families. (**A**) THT_2008 comparison between the DxP control and the GM-3341 family in the PT132 trial in the East Estate; (**B**) FL comparison between the DxP control and the GM-3341 family in the PT132 trial in the East Estate; (**C**) THT_2008 comparison between the DxP control and the GM-DAV-3461 family in PT138 in the North Estate; (**D**) FL comparison between the DxP control and the GM-DAV-3461 family in PT138 in the North Estate. The trait comparisons were analyzed based on two-sample *t* test at the *p* = 0.05 threshold.

Sixteen traits related to oil yield, THT and FL were recorded in the GM-3341 *dura* family (*n* = 107 palms) and the GM-DAV-3461 *tenera* family (*n* = 70 palms) located in PT132 in the East Estate and PT138 in the North Estate, respectively (Table S3). A commercial DxP control was also included in each trial and served as a control for comparison of traits using a two-sample *T* test at the *p* = 0.05 threshold. After removal of the outliers, the 16 traits followed normal distributions at the *p* = 0.05 threshold and the dataset was subjected to subsequent analyses. The GM-3341 family produced 19.50 ± 5.00 kg/palm/year of O/P, which was lower than the 26.25 ± 7.44 kg/palm/year produced by the GM-DAV-3461 family. Poorer average performance in both FFB (103.37 ± 21.21 kg/palm/year) and O/B (18.72 ± 2.70%) in the GM-3341 family were the main contributors, compared to 125.90 ± 24.49 kg/palm/year of average FFB and 20.29 ± 3.90% of average O/B in the GM-DAV-3461 family. Shell thickness variation within *tenera* was measured by the shell-to-fruit ratio (S/F). As expected, the GM-3341 family had a thicker shell (average S/F = 30.97 ± 3.24%) than that of the GM-DAV-3461 family (average S/F = 15.59 ± 2.82%), with the GM-3341 being purely *dura* shell-types and the GM-DAV-3461 being purely the thinner *tenera* hybrid. In terms of average fruit-to-bunch ratio (F/B), the GM-3341 family and the GM-DAV-3461 recorded 63.60 ± 4.71% and 60.45 ± 5.63%, respectively, which were within the normal range, i.e., 60–65% [13]. Trunk height and FL were measured twice, once in 2008 (as THT_2008) and also in 2014 (as THT_2014) at PT132 trial, whereas only a single measurement was done at the PT138 trial in 2008 (Table S3). The GM-3341 family at the PT132 trial with a mean THT_2008 = 145.07 cm and mean FL = 534.53 cm were significantly shorter than the DxP control, which had means for THT_2008 = 213.02 cm and FL = 554.83 cm at the *p* = 0.05 threshold for both (Figure 1A,B). The same result was observed in the PT138 trial, where the GM-DAV-3461 family had means for THT_2008 = 206.37 cm and FL = 565.34 cm and were significantly shorter than the DxP control with means for THT_2008 = 266.90 cm and FL = 584.30 cm at the same *p* threshold (Figure 1C,D).

### 3.2. Pearson Correlation among Traits

The correlations among all the 16 traits of the GM-3341 family are illustrated in Table S4. As mentioned, O/P is a derivative trait from multiplying FFB and O/B per palm, thus significant correlations with FFB (correlation coefficient, *r* = 0.619; *p* = 0.000) and O/B (*r* = 0.332; *p* = 0.007) were observed. The same effects applied to the component traits of FFB and O/B. Fresh fruit bunch weight significantly correlated with BNO (*r* = 0.824; *p* = 0.000) and average BWT (*r* = 0.253; *p* = 0.012), while O/B significantly correlated with F/B (*r* = 0.473; *p* = 0.000), mesocarp-to-fruit ratio (M/F) (*r* = 0.490; *p* = 0.000) and oil-to-wet mesocarp (O/WM) (*r* = 0.444; *p* = 0.000). In addition, oil palm fruitlets are mostly made up of an outer layer of mesocarp and a central nut consisting of a shell-coated kernel. Hence, each of the fruitlet components is actually a proportion of the mean fruit weight (MFW) and this was reflected in the significant correlations among MFW, S/F, M/F and the kernel-to-fruit ratio (K/F). A significant correlation was also detected between independent oil yield-related traits such as BNO with F/B (*r* = -0.262; *p* = 0.036). For vegetative traits, there was a correlation between FL and THT (THT_2008, *r* = 0.270; *p* = 0.008 and THT_2014, *r* = 0.301; *p* = 0.009) (Table 1, Table S4). Interestingly, THT recorded in 2008 and 2014 was not highly correlated (*r* = 0.595; *p* = 0.000), suggesting the non-linear THT increment of oil palm over the growing cycle. THT_2008 correlated better with yield-related traits including O/P, FFB, BNO and BWT, compared to THT_2014 and FL. This is likely to be before (THT_2008) and after (THT_2014) palm-to-palm competition becomes important.

### 3.3. Construction of Linkage Maps

**Figure 2:**
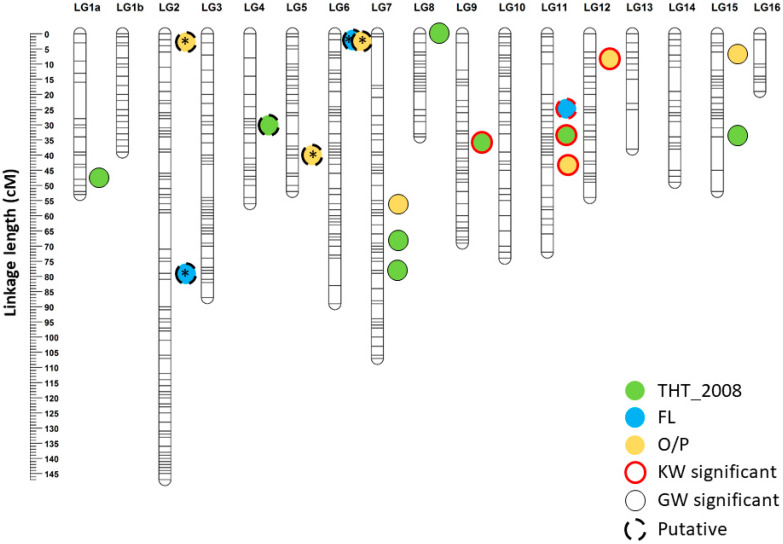
Distribution of quantitative trait loci (QTLs) for trunk height in 2008 (THT_2008), frond length (FL) and total oil per palm (O/P) on a linkage map of the GM-3341 family. KW significant—Kruskal-Wallis at the *p* = 0.005 threshold; GW significant—genome-wide logarithm of odds, to the base 10 (LOD) = 4.0 threshold; putative—2.4 ≤ LOD < 4.0 threshold; * non-cofactor.

The GM-3341 family, as a mapping population was genotyped using 200,000 SNPs. Of these, 33,190 SNPs were considered informative for linkage mapping and were successfully binned into linkage groups (LGs) that were consistent with 16 chromosome pairs of oil palm genome (2n = 32). The constructed linkage map with 33,190 SNPs spanned a cumulative length of 1618.51 centiMorgan (cM) with an average mapping interval of 0.05 cM and was termed the “full-density” map (data not shown). For QTL localization, a subset of 588 SNPs with an average mapping interval of 5 cM were coupled with 67 informative SSRs (Tables S1 and S2) to build a linkage map with reduced density. From this, 565 markers with ≤5% missing data were linked as a linkage map that had a cumulative length of 1093.51 cM and a lower average mapping interval of 2.01 cM (Figure 2, Table 2). The number of LGs, however, increased to 17, because LG1 fragmented into two, namely, LG1a and LG1b. The marker positions between full and reduced-density linkage maps were compared and were generally in good agreement. The latter map was then adopted for estimating an average recombination rate throughout the genome, which was 1.74 cM/Mb, and which ranged from 0.63 cM/Mb (LG1b) to 3.24 cM/Mb (LG10).

### 3.4. Mapping of QTLs for THT, FL and O/P Traits

The QTL mapping for THT and correlated traits started with single marker testing on 655 linked markers using the ranking sum test of KW. A total of 49 significant markers were detected to be effecting THT_2008 (30 markers), THT_2014 (1 marker), FL (13 markers) and O/P (5 markers) at the *p* = 0.005 threshold (Table S5). Subsequently, the lower-density linkage map of the GM-3341 family was used to conduct IM. The detected IM-based QTLs were fixed as cofactors for MQM analyses. LOD = 4.0 was set as the genome-wide significance threshold based on permutation tests with 1000 iterations at α = 0.05, whereas the QTLs within 2.5 ≤ LOD <4.0 were termed “putative”. First of all, no significant QTL for THT_2014 was found in IM and MQM. As shown in Figure 2, however, genome-wide significant QTLs for THT_2008 were detected on LG1a, LG7, LG8, LG9, LG11 and LG15, explaining 7.4–17.0% of the trait variation each (Figure 2, Table 3). Another putative QTL for the same trait was located on LG4 with 4.3% of the variation explained. As for FL, four putative QTLs on LG2 and LG6 and LG11 that explained 10.2–16.5% of the variation were found. Three of the QTLs on LG2 (rs795951455) and LG6 (rs795993306 and ss1810567844) emerged as non-cofactor peaks when rs795990373 on LG11 was fixed as a cofactor. Similarly, by fixing the four genome-wide significant QTLs for O/P residing at 56.03 cM on LG7, 43.79 cM on LG11, 34.73 cM on LG12 and 16.25 cM on LG15 as cofactors, three putative QTLs emerged on LG2, LG6 and LG5. The O/P variation explained by these QTLs ranged from 6.0–28.5%.

### 3.5. Cumulative Effects of Quantitative Trait Allele Analysis

**Figure 3:**
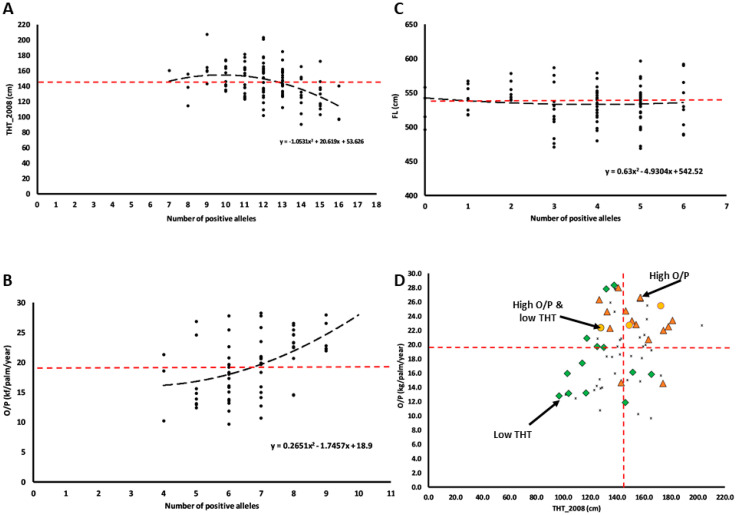
Cumulative effects of quantitative trait alleles for (**A**) THT_2008, (**B**) O/P, (**C**) FL traits, and (**D**) the application for selection of THT_2008 and O/P traits in the GM-3341 Family. Red dotted line—mean THT_2008 (145.07 cm), mean O/P (19.50 kg/palm/ year) and mean FL (534.53 cm); orange dot—High O/P and low THT_2008, red triangle—High O/P, green diamond—Low THT_2008; multiple trait selection in Figure 3D based on threshold numbers of positive alleles for THT_2008 = 13 and for O/P = 7.

In this analysis, we adopted 16, 9 and 6 positive alleles for THT_2008, O/P and FL, respectively. Only THT_2008 and O/P traits fit well into quadratic relationships between the number of the positive QTL alleles and trait variation, but in antagonistic directions (Figure 3A–C). The palms carrying more than 13 positive alleles for THT_2008 trait were mainly shorter than the population mean = 145.07 cm, whereas those with more than 7 positive alleles for O/P trait mainly yielded higher than the population mean = 19.50 kg/palm/year. Hence, the number of positive alleles for respective THT_2008 and O/P were used for multiple trait selection (Figure 3D). We found that 77% and 86% of the selected palms had shorter THT and higher O/P than the population mean, suggesting that the single trait selection method was effective. For multiple trait selection, by contrast, only one palm in the GM3341 family with low THT and high O/P (the orange dot shown in Figure 3D) was identified using the QTL markers.

## 4. Discussion

Oil palm breeding in Southeast Asia predominantly started with the Deli *dura*, which is believed to be derived from four palms planted in the Bogor Botanical Garden, Indonesia in 1848 [25,26]. The Deli *dura* were subsequently distributed to various breeding programs, each of which stressed different selection approaches over generations and caused separation between subpopulations, which are known as “breeding populations of restricted origin” (BPRO) [27]. However, these breeding programs still share the same overall objective, that is, superior oil yield, which has been achieved by different selection for traits. In addition, understanding the co-dominant inheritance of the shell thickness gene [28] also paved the way for exploitation of the superior oil content of the thin-shelled *tenera*, which was commercially produced from hybridization between thick-shelled Deli *dura* and shell-less AVROS *pisifera*. At the beginning of the 1960s, planters in the Southeast Asia region successfully realized a 30% increment in oil yield per hectare as *tenera* planting materials replaced the original Deli *dura* materials [13,29]. In this study, we also observed a 34.6% O/P increment from the GM-3314 *dura* family (19.50 ± 5.00 kg/palm/year) to the DAV-GM-3461 *tenera* family (26.25 ± 7.44 kg/palm/year) (Table S3).

Nevertheless, the oil palm industry in Malaysia started to experience labor shortages, particularly for FFB and loose fruit collection, around a decade ago and the problem is escalating. Hence, the breeding objective of superior oil yield is being expanded to include traits, such as low THT and compactness to improve harvest and management efficiency. In this study, low THT and short frond length within the GM-3341 family and its half-sib GM-DAV-3461 family was found to be heritable from GM *dura* and DAV *pisifera* (Figure 1). This finding supports the idea that both traits can actually be improved through hybridization between GM and DAV origins to produce short or even compact *tenera* palms, compared to using a single genetic donor of GM origin as reported earlier [11]. The robustness of trait data was verified based on significant correlations between the derivative traits and associated component traits of the GM-3341 family at the *p* = 0.05 threshold (Table S4). Meaningful biological interpretation was also performed based on the correlations between independent traits. For example, an inverse correlation between BNO and F/B (*r* = −0.262; *p* = 0.036) is likely to be related to the pollination ability of weevils (*Elaeidobius kamerunicus*), which is reflected in the number of insects per receptive female inflorescence per hectare [30]. Decline in “pollination force”’ can happen if many receptive female inflorescences are developed in an area with a fixed population of weevils. In such scenarios, the palms still can produce more BNO, but a smaller number of flowers on these female inflorescences were pollinated to form fruitlets, leading to lower FFB. Bunches with more than 60% F/B are considered normal [30,31], so, both the GM-3341 family and the GM-DAV-3461 family did not encounter a fruit set problem.

In the GM-3341 family, trunk growth was found to be non-linear across different ages based on a moderate correlation between THT_2008 (seven years after planting) and THT_2014 (13 years after planting) (*r* = 0.595; *p* = 0.000) (Table 1). This finding agrees with previous studies of trunk growth in oil palm, which are generally affected by three main factors, i.e., palm age, genotype and environmental conditions (such as agronomic practices and soil types) [32]. In the first 3 years, trunk growth is usually negligible [33] and then becomes significant at six years onwards [34]. Furthermore, many studies also reported that THT does increase with planting density, but the effect only becomes observable at 9 years after planting, which is when the canopy starts to overlap [35,36]. The mature palms tend to grow taller to compete for sunlight, so the genotype effect on THT is no longer as clear at that stage. Taller palms will also capture more sunlight, so small differences in height may lead to large differences in FFB. This may have complicated the QTL detection for THT_2014 in IM and MQM. Palm-to-palm competition might affect the yield mechanism in these palms as well, based on the missing correlations between THT_2014 and yield traits (O/P, FFB, BNO and BWT). It is worth noting that the yield recording in this study was conducted around 7 years after planting, so is unlikely to include the effects of competition between palms.

Oil palm breeders have attempted to stack superior O/P, low THT and short FL to produce compact palms. In most breeding programs, multiple trait integration becomes a real challenge when desirable traits are antagonistic to each other. Such a phenomenon is common in oil palm breeding for high-yielding, compact and low THT palms. We noticed that THT_2008 was significantly correlated to O/P (*r* = 0.294) and FL (*r* = 0.270), which is undesirable from a breeding perspective (Table 1, Table S4). These correlations seem to be biologically sensible in that vigorous vegetative growth of fronds and trunk height help to capture more sunlight for photosynthesis. However, it is not simple as that. In Arabidopsis, the relationship between leaf area growth and plant biomass was reported to be non-linear; it depends on how carbon assimilates produced from photosynthesis are partitioned into new leaf area, roots, reproduction and respiration [37]. In fact, the same findings for oil palm were also reported three decades ago [38,39]. Photosynthesis rate increases in response to sink demand in the crop. Extra assimilates will only be allocated to FFB production (effectively oil yield) after the demand for vegetative growth and maintenance is satisfied [34]. Thus, the moderate correlation between THT_2008 and O/P raises the possibility of selecting palms that buck this trend and have a higher proportion of dry matter in fruit bunches. However, when dealing with multiple complex traits, breaking linkage drag in perennial oil palm through conventional breeding methods will always be hindered by small progeny size with 16–96 palms per cross. With a planting density of 136–146 palms per hectare, the field evaluation of a large number of progenies is too costly and labor intensive—this is where MAS can help.

By using the OP200K genotyping array [12], the GM family with 33,190 linked markers was more genetically diverse than the reported commercial Deli × AVROS family with 27,890 linked markers [40], which presumably reflects the effect of breeding selection pressure in the latter population. High-density linkage maps are known to be a powerful way to improve physical genome assemblies. For instance, a further 311 Mb of scaffolds were successfully anchored to the pseudo-chromosomes of oil palm based on the Deli × AVROS linkage map [40]. The genome of melon (*Cucumis melo* L.) double haploid line DL92 was also extended with 354.8 Mb of sequence using a linkage map with an average mapping interval of 1.99 cM [41]. Nonetheless, QTL localization on a high-density linkage map using the MapQTL^®^ 6 program is always constrained by insufficient degrees of freedom to accommodate many markers (typically > 600) due to uninformative segregation types in cross-pollinating (CP) populations [24], which is common in oil palm. Hence, we only selected markers at around 5 cM apart to construct a lower-density linkage map with 565 linked markers that spanned a cumulative length of 1093.51 cM with an average mapping interval of 2.01 cM, but with good genome coverage (Figure 2, Table 2). The marker order in both maps were highly correlated but the mapping interval was smaller than 5 cM, as expected when a degree of conflicting data is removed. Inflated map length and orientation errors are likely to be reduced after removing some markers with incorrect SNP calling. The genome-wide recombination rate of 1.74 cM/mb calculated in the GM genome was close to the Deli × AVROS genome (1.8 cM/Mb) [40] and the date palm genome (*Phoenix dactylifera*) (1.9 cM/Mb) [42], suggesting this is a reasonable range for palm species.

For QTL mapping, double recombination should rarely occur in the lower-density linkage map, which is still much denser than the 10 cM threshold [43]. Linkage group 1 fragmented into LG1a and LG1b in the lower-density map. In fact, this did not restrict the analysis, because all LGs are traceable to the 16 homologous chromosomes of the reference genome [23]. We preferred MQM analysis because it is a single dimensional search over the genome to test a single segregating QTL as in IM, while simultaneously fitting the selected cofactor, under both the null-hypothesis (H_0_) and the alternative hypothesis (H_1_). With this approach, the cofactors can reduce the residual variance, therefore, if a QTL explains a large proportion of the total variance, the linked markers used as cofactors in subsequent MQM analyses can significantly improve the power for identifying new segregating QTLs [24]. This method thus enabled detection of new QTLs for THT_2008, O/P and FL traits after fixing the major QTLs on LG9, LG11 and LG12 as cofactors (Figure 2). Interestingly, we found that ss1810516343 as the major QTL for THT_2018 was closely flanked by putative QTL locus rs795990373 for FL and genome-wide significant rs796015770 for O/P on LG9. This marker arrangement strongly reflects the correlation of THT_2008 with O/P and FL, suggesting a possible pleotropic effect. In fact, the effects of reduced height on increased yield are seen in other crops. A good example is wheat, where the famous Reduced Height (*Rht*) gene has a pleotropic effect, decreasing plant height and increasing grain yield [44]; the subsequent discovery of *Rht-B1b* and *Rht-D1b* successfully improved seed yield by 5–10% with a 20% stem height reduction [45].

This study provides initial QTL locations to enable identification of causal genes, particularly for THT and O/P traits. For this purpose, the QTLs that were genome-wide and KW significant should be given the highest priority (Table 2). The GM-based QTLs for THT identified in this study are in different locations from previous studies based on Deli *dura* × DAV origin [46] and *Dura* × Ghana origin [47], which reveals the possible effects of different genes or gene paralogues across the genomes derived from different origins. Consequently, the application of the current set of QTL genes for height may remain origin specific, unless the genetic causality is fully understood. To prepare for MAS in the GM origin, the cumulative effects of quantitative trait alleles for THT_2008, O/P and FL were used as a model to determine the effects of different numbers of positive alleles for the best selection response. The results show that single trait selections for THT_2008 and FL based on 13 and seven positive alleles were promising for the former but not for FL (Figure 3). The FL variation did not significantly deviate from the population mean (534.53 cm) regardless of how many positive alleles were carried; thus, the trait was omitted for multiple locus selection. Using the same thresholds, we identified only 1% of the GM3341 family with low THT and high O/P to be selected for progeny testing with DAV *pisifera* palms. In a multiple trait integration program in oil palm, this probability of success makes it is close to “impossible” to break the linkage drag, especially if the current breeding approach of using a limited family size (usually not more than 96 palms assessed per cross) remains unchanged. Before field planting, breeders can plant all 1000–1500 seedlings per cross in the nursery and select for the best 100 palms using the markers derived from this research, for field planting for the next breeding cycle. A 1% selection would provide 10–15 palms containing the desired alleles and multiple bunches of the same cross would provide large enough families to field select for additional variation beyond the selected loci.

## 5. Conclusions

The reduced THT and FL traits from GM and DAV origins have been determined, and show great potential for selective breeding. Further understanding of the correlations between agronomic traits and the effects of the cumulative positive alleles of QTLs pave the way for breeders to conduct multiple trait integration through MAS programs, particularly to produce high-yielding short or compact oil palms. With a 1% probability of breaking linkage drag, future trials need to be saturated with elite palms selected from a large starting population. However, non-linear THT growth across developmental stages should also be taken cautiously, depending on the breeding objectives.

## Figures and Tables

**Table 1:** Pearson correlation of trunk height traits, frond length and oil yield-related traits of the GM-3341 family.

Trait	Trait Abbreviation	THT_2008	THT_2014
Fresh fruit bunch	FFB/kg	0.423 *	0.242 *
Bunch number	BNO	0.364 *	0.119
Bunch weight	BWT	0.248 *	0.156
Fruit-to-bunch ratio	F/B	−0.055	−0.015
Mean fruit weight	MFW	−0.068	0.107
Mesocarp-to-fruit ratio	M/F	−0.150	0.103
Shell-to-fruit ratio	S/F	0.156	−0.153
Kernel-to-fruit ratio	K/F	0.033	0.043
Oil-to-dry mesocarp ratio	O/DM	0.237	0.216
Oil-to-wet mesocarp ratio	O/WM	−0.038	−0.042
Oil-to-bunch ratio	O/B	0.126	0.069
Kernel-to-bunch ratio	K/B	0.081	−0.005
Total oil per palm	O/P	0.294 *	0.165
Frond length	FL	0.270 *	0.301 *
Trunk height in 2008	THT_2008	NA	0.595 *
Trunk height in 2014	THT_2014	NA	NA

* Significant correlation at the *p* = 0.05 threshold; NA—not applicable.

**Table 2:** Linkage length and mapping interval across linkage groups in the GM-3341 family.

No.	Group	Linkage Length (cM)	No. of Linked Markers	Mapping Interval (cM)	No. of Markers in the Physical Map	Linkage Length of Common Markers (cM)	Physical Length (Mb)	Average Recombination Rate (cM/Mb)
1	LG1a	53.44	20	2.67	10	53.44	63.71	0.84
2	LG1b	38.99	20	1.95	18	38.99	61.63	0.63
3	LG2	147.43	73	2.02	36	147.43	64.78	2.28
4	LG3	86.94	48	1.81	27	86.94	58.66	1.48
5	LG4	55.97	32	1.75	9	55.97	34.73	1.61
6	LG5	52.11	34	1.53	20	52.11	37.29	1.40
7	LG6	88.89	55	1.62	22	83.33	41.90	1.99
8	LG7	107.34	49	2.19	27	105.61	42.27	2.50
9	LG8	33.67	28	1.20	22	33.67	39.98	0.84
10	LG9	69.28	38	1.82	23	68.16	34.66	1.97
11	LG10	74.31	35	2.12	19	74.31	22.94	3.24
12	LG11	72.36	37	1.96	13	72.36	26.05	2.78
13	LG12	54.00	28	1.93	26	54.00	26.63	2.03
14	LG13	37.90	11	3.45	7	23.12	25.51	0.91
15	LG14	49.50	23	2.15	18	49.50	23.78	2.08
15	LG15	51.95	22	2.36	16	45.24	22.25	2.03
17	LG16	19.43	12	1.62	9	16.43	17.05	0.96
	GW	1093.51 *	565 *	2.01 *	322 *	1060.61 *	643.82 *	1.74 **

* Sum total; ** average value; GW—genome-wide; LG—linkage map; cM—centiMorgan; Mb—Megabase; each LG identified as a chromosome of the published genome [23].

**Table 3:** Multiple QTL mapping and Kruskal-Wallis test for trunk height recorded in 2008 (THT_2008), frond length (FL) and total oil per palm (O/P) traits in the GM-3341 family.

Trait	Linkage Group	Cofactor	Peak LOD	Peak Position (cM)	Trait Variation Explained (%)	KW
THT_2008	1a	rs795987799	4.55	48.04	7.6	
	4	rs795956088	2.67	30.22	4.3	
	7	rs796035893	6.65	68.67	11.7	
	7	ss1810504935	6.79	78.32	12.0	
	8	rs795967221	5.02	0.00	8.5	
	9	rs796032489	4.42	35.56	7.4	*****
	11	ss1810516343	9.11	33.55	17.0	****
	15	rs795995560	5.99	33.79	10.4	
O/P	2	DA290 ^NC^	3.74	81.14	7.6	
	5	rs796007949 ^NC^	2.88	39.55	6.0	
	6	DA358 ^NC^	3.02	88.89	6.3	
	7	ss1810302303	4.09	56.03	10.9	
	11	rs796015770	4.56	43.79	12.4	****
	12	rs796017329	8.13	34.73	25.2	****
	15	rs795992318	8.92	16.25	28.5	
FL	2	rs795951455 ^NC^	2.69	79.40	10.2	
	6	rs795993306 ^NC^	3.11	2.26	11.7	
	6	ss1810567844 ^NC^	3.11	2.38	11.7	
	11	rs795990373	3.73	24.55	16.5	******

THT_2008—Trunk height in 2008, THT_2014—Trunk height in 2014, O/P—Total oil per palm, ^NC^ non-cofactor, cM—centiMorgan, KW—Kruskal-Wallis statistics, **** 0.005 significance level, ***** 0.001 significance level, ****** 0.0005 significance level.

## Supplementary Materials

The following are available online at https://www.mdpi.com/2073-4425/11/7/826/s1, Table S1: The 655 selected markers (588 SNPs and 67 SSRs) for linkage map construction. Table S2: Sequences of 67 SSR primer pairs. Table S3: Descriptive statistics of oil yield, frond length and trunk height traits. Table S4: Person correlation among the 16 traits of the GM-3341 family. Table S5: Kruskal-Wallis analyses of trait-affecting markers. All SNPs of the OP200K genotyping array have been deposited in dbSNP under the handle of SDTC_BB with NCBI submitted SNP (ss) accession numbers of 1810069240-1810592638.
