# Supplementary material for: Major QTLs for Trunk Height and Correlated Agronomic Traits Provide Insights into Multiple Trait Integration in Oil Palm Breeding
Source: Genes (Basel). 2020 Jul 21;11(7):826. doi: 10.3390/genes11070826 (PMC7397176; doi:10.3390/genes11070826)
Supplement: Supplementary file 1 [file genes-11-00826-s001.zip › Revised Supplementary Materials/Table S3.docx]

Table S3. Descriptive statistics of oil yield, frond length and trunk height traits.

| **Trait** | **Abbreviation** | **Unit** | **GM-3341** | | | | **GM-DAV-3461** | | | |
| --- | --- | --- | --- | --- | --- | --- | --- | --- | --- | --- |
|  |  |  | **Mean** | **Median** | **SD** | **CV%** | **Mean** | **Median** | **SD** | **CV%** |
| Fresh fruit bunch | FFB | kg/palm/year | 103.37 | 104.35 | 21.21 | 20.52 | 125.90 | 129.15 | 24.49 | 19.45 |
| Bunch number | BNO | No./palm/year | 9.29 | 9.20 | 2.29 | 24.62 | 13.28 | 13.40 | 2.43 | 18.32 |
| Bunch weight | BWT | kg/palm/year | 10.97 | 11.05 | 1.42 | 12.92 | 9.34 | 9.30 | 1.41 | 15.05 |
| Fruit-to-bunch ratio | F/B | % | 63.60 | 63.10 | 4.71 | 7.41 | 60.45 | 60.70 | 5.63 | 9.31 |
| Mean fruit weight | MFW | g | 10.33 | 10.20 | 2.27 | 21.95 | 7.64 | 7.30 | 1.84 | 24.10 |
| Mesocarp-to-fruit ratio | M/F | % | 61.91 | 62.65 | 4.22 | 6.82 | 74.44 | 74.75 | 4.44 | 5.96 |
| Shell-to-fruit ratio | S/F | % | 30.97 | 30.60 | 3.24 | 10.46 | 15.69 | 16.20 | 2.82 | 17.95 |
| Kernel-to-fruit ratio | K/F | % | 6.93 | 6.90 | 1.50 | 21.67 | 9.41 | 9.30 | 2.28 | 24.27 |
| Oil-to-dry mesocarp ratio | O/DM | % | 76.61 | 76.95 | 2.62 | 3.41 | 73.98 | 74.60 | 3.43 | 4.64 |
| Oil-to-wet mesocarp ratio | O/WM | % | 48.98 | 48.90 | 4.01 | 8.32 | 45.17 | 45.80 | 6.69 | 14.81 |
| Oil-to-bunch | O/B | % | 18.72 | 18.95 | 2.70 | 14.41 | 20.29 | 20.30 | 3.90 | 19.23 |
| Kernel-to-bunch ratio | K/B | % | 4.11 | 4.10 | 1.03 | 25.13 | 5.47 | 5.40 | 1.39 | 25.49 |
| Total oil per palm | O/P | kg/palm/year | 19.50 | 19.70 | 5.00 | 25.66 | 26.25 | 26.30 | 7.44 | 28.33 |
| Frond length | FL | cm | 534.53 | 539.10 | 28.64 | 5.36 | 565.34 | 566.10 | 27.01 | 4.78 |
| Trunk height in 2008 | THT_2008 | cm | 145.07 | 143.10 | 23.07 | 15.90 | 206.37 | 207.35 | 35.87 | 17.38 |
| Trunk height in 2014 | THT_2014 | cm | 355.80 | 356.35 | 55.38 | 15.56 | nil | nil | nil | nil |

SD – standard deviation, CV% - coefficient of variance. The 16 assayed traits were normally distributed based on Anderson-Darling normality test at the *p* = 0.05 threshold.
